# Construction and Evaluation of Multiple Radiomics Models for Identifying the Instability of Intracranial Aneurysms Based on CTA

**DOI:** 10.3389/fneur.2022.876238

**Published:** 2022-04-11

**Authors:** Ran Li, Pengyu Zhou, Xinyue Chen, Mahmud Mossa-Basha, Chengcheng Zhu, Yuting Wang

**Affiliations:** ^1^Department of Radiology, Sichuan Provincial People's Hospital, University of Electronic Science and Technology of China, Chengdu, China; ^2^Computed Tomography Angiography Collaboration, Siemens Healthineers, Chengdu, China; ^3^Department of Radiology, University of Washington School of Medicine, Seattle, WA, United States

**Keywords:** intracranial aneurysm, computed tomography angiography, machine learning, Radiomics, risk assessment

## Abstract

**Background and Aims:**

Identifying unruptured intracranial aneurysm instability is crucial for therapeutic decision-making. This study aims to evaluate the role of Radiomics and traditional morphological features in identifying aneurysm instability by constructing and comparing multiple models.

**Materials and Methods:**

A total of 227 patients with 254 intracranial aneurysms evaluated by CTA were included. Aneurysms were divided into unstable and stable groups using comprehensive criteria: the unstable group was defined as aneurysms with near-term rupture, growth during follow-up, or caused compressive symptoms; those without the aforementioned conditions were grouped as stable aneurysms. Aneurysms were randomly divided into training and test sets at a 1:1 ratio. Radiomics and traditional morphological features (maximum diameter, irregular shape, aspect ratio, size ratio, location, etc.) were extracted. Three basic models and two integrated models were constructed after corresponding statistical analysis. Model A used traditional morphological parameters. Model B used Radiomics features. Model C used the Radiomics features related to aneurysm morphology. Furthermore, integrated models of traditional and Radiomics features were built (model A+B, model A+C). The area under curves (AUC) of each model was calculated and compared.

**Results:**

There were 31 (13.7%) patients harboring 36 (14.2%) unstable aneurysms, 15 of which ruptured post-imaging, 16 with growth on serial imaging, and 5 with compressive symptoms, respectively. Four traditional morphological features, six Radiomics features, and three Radiomics-derived morphological features were identified. The classification of aneurysm stability was as follows: the AUC of the training set and test set in models A, B, and C are 0.888 (95% CI 0.808–0.967) and 0.818 (95% CI 0.705–0.932), 0.865 (95% CI 0.777–0.952) and 0.739 (95% CI 0.636–0.841), 0.605(95% CI 0.470–0.740) and 0.552 (95% CI 0.401–0.703), respectively. The AUC of integrated Model A+B was numerically slightly higher than any single model, whereas Model A+C was not.

**Conclusions:**

A radiomics and traditional morphology integrated model seems to be an effective tool for identifying intracranial aneurysm instability, whereas the use of Radiomics-derived morphological features alone is not recommended. Radiomics-based models were not superior to the traditional morphological features model.

## Introduction

Intracranial aneurysms have a prevalence of 3–5% in the adult population worldwide (1, 2), however, the annual rupture incidence is 9 in 100,000 cases (2). Ruptured intracranial aneurysms with subarachnoid hemorrhage cause a mortality rate approaching 50% (3, 4). In order to prevent the catastrophic consequences of aneurysm rupture, predicting intracranial aneurysm rupture risk is a need.

Current risk stratification of intracranial aneurysms relies heavily on traditional factors such as aneurysm size, morphology, and certain demographic risk factors (5–7). Although aneurysm size on imaging is one of the important features regarding rupture risk, many aneurysms rupture at diameters <7 mm (8), the threshold for surgical intervention proposed by The International Study of Unruptured Intracranial Aneurysms (ISUIA) (9). Therefore, other aneurysm features related to rupture risk have been explored. Advanced morphological parameters have drawn great attention and multiple mathematical calculational methods have been developed to define them. Proposed features included NSI (non-sphericity index), UI (undulation index), EI (ellipticity index) and AR (aspect ratio) (10). Manual extraction of these features, however, was limited. The quantitative features and patterns of target lesions in medical images can be extracted automatically with high throughput (11).

Radiomics is an emerging analytical method with the ability to screen thousands of features through specific algorithms. The systematic approach by Radiomics to extract, screen, and verify more advanced parameters pixel-by-pixel has the potential to reveal new biomarkers for aneurysm risk prediction, which has been proved valuable in medical fields especially in tumor research in recent years (11), but its application in intracranial aneurysms remains preliminary. Some recent Radiomics studies explored DSA (digital subtraction angiography) data to identify aneurysm instability (12). As a technique, however, the invasiveness of DSA limits its use, especially in serial follow-up of aneurysms. CTA is an economical and non-invasive examination and the first-line imaging modality for aneurysm characterization, assessment and longitudinal evaluation. Ou et al. used Radiomics of CTA data to discriminate ruptured and unruptured aneurysms to characterize aneurysm instability (13), however, a limitation of this approach is cerebral aneurysm morphologies differ significantly before and after rupture (14), and the features of ruptured aneurysms may not accurately represent those of the unstable aneurysms before the rupture event. A meta-analysis found that the risk of rupture of symptomatic aneurysms was 4.4 times higher than that of asymptomatic aneurysms (15). This subgroup of symptomatic and unstable aneurysms, however, have not been included in Radiomics analysis yet. Multiple studies suggested that growing aneurysms have significantly increased risk of aneurysm rupture (7, 16, 17), but previous studies employing Radiomics barely included longitudinal data of cerebral aneurysms. In addition, a direct comparison between Radiomics analysis and established traditional morphological features are lacking in the literature (13, 18, 19).

This study aims to employ more comprehensive criteria for unstable unruptured intracranial aneurysms, evaluate the performance of Radiomics for aneurysm risk stratification, and compare that with the performance of traditional morphological features.

## Materials and Methods

### Study Population

Consecutive patients imaged at Sichuan Provincial People's Hospital from November 2015 to December 2019 who were diagnosed as having intracranial aneurysms on CTA were retrospectively reviewed. Among these patients, the study population was selected based on the following inclusion criteria: (1) confirmed diagnosis of one or more unruptured saccular cerebral aneurysms at baseline by CTA (positive diagnosis in the clinical record by experienced radiologist); (2) aneurysm maximum diameter >2 mm and lesion morphology that is differentiable from infundibulum; (3) image quality allowed detailed depiction of aneurysm morphology and parent vessels, and was sufficient for the segmentation without substantial artifacts. Exclusion criteria: (1) fusiform, dissecting, traumatic, or infectious aneurysms; (2) the presence of concomitant cerebral vascular diseases, such as high-flow arteriovenous malformation, Moyamoya disease, arteriovenous fistula, dissection, vasospasm and inflammatory vasculopathy; (3) acute aneurysm rupture with presence of subarachnoid hemorrhage; (4) aneurysm endovascular treatment prior to CTA imaging.

Included aneurysms were divided into two groups according to their stability, following the criteria of a previous study (12) and supported by multiple studies (20, 21). The positive group (unstable aneurysms, considered with high risk of rupture) consists of 3 subgroups. The first subgroup is aneurysms with a near-term rupture event that occurred after CTA image acquisition (within 4 days). This category included patients who presented with sentinel headaches, negative non-contrast CT head, and presence of aneurysm on CTA. Aneurysm rupture was subsequently diagnosed on follow-up non-contrast CT head, with presence of hyperdense subarachnoid hemorrhage. We did not include ruptured aneurysms because aneurysm morphology changes after rupture (14). The second subgroup in the positive group is growing aneurysms. Growing aneurysms were identified on sequential angiographic imaging where the maximum diameter increased by more than 1 mm between two imaging exams according to the ELAPSS criteria (22). The third subgroup is symptomatic aneurysms, defined as patients with aneurysms causing compressive symptoms (ex. oculomotor nerve compression confirmed intraoperatively) (12). Other less specific symptoms including dizziness and headaches which could not be definitively attributed to aneurysm were not included in the symptomatic group. The negative group (stable aneurysms, considered to have low rupture risk) included incidentally discovered asymptomatic aneurysms or those undergoing follow-up without rupture or growth. When multiple aneurysms were identified, those that met the inclusion and exclusion criteria were included in the analysis, and each aneurysm was assessed and classified into the positive or negative group, respectively. For instance, if a patient with multiple aneurysms had near-term SAH and underwent intervention, the ruptured aneurysm was classified as previously unstable (at the time of the prior CTA), and the remaining unruptured aneurysms were classified as stable.

**Figure 1:**
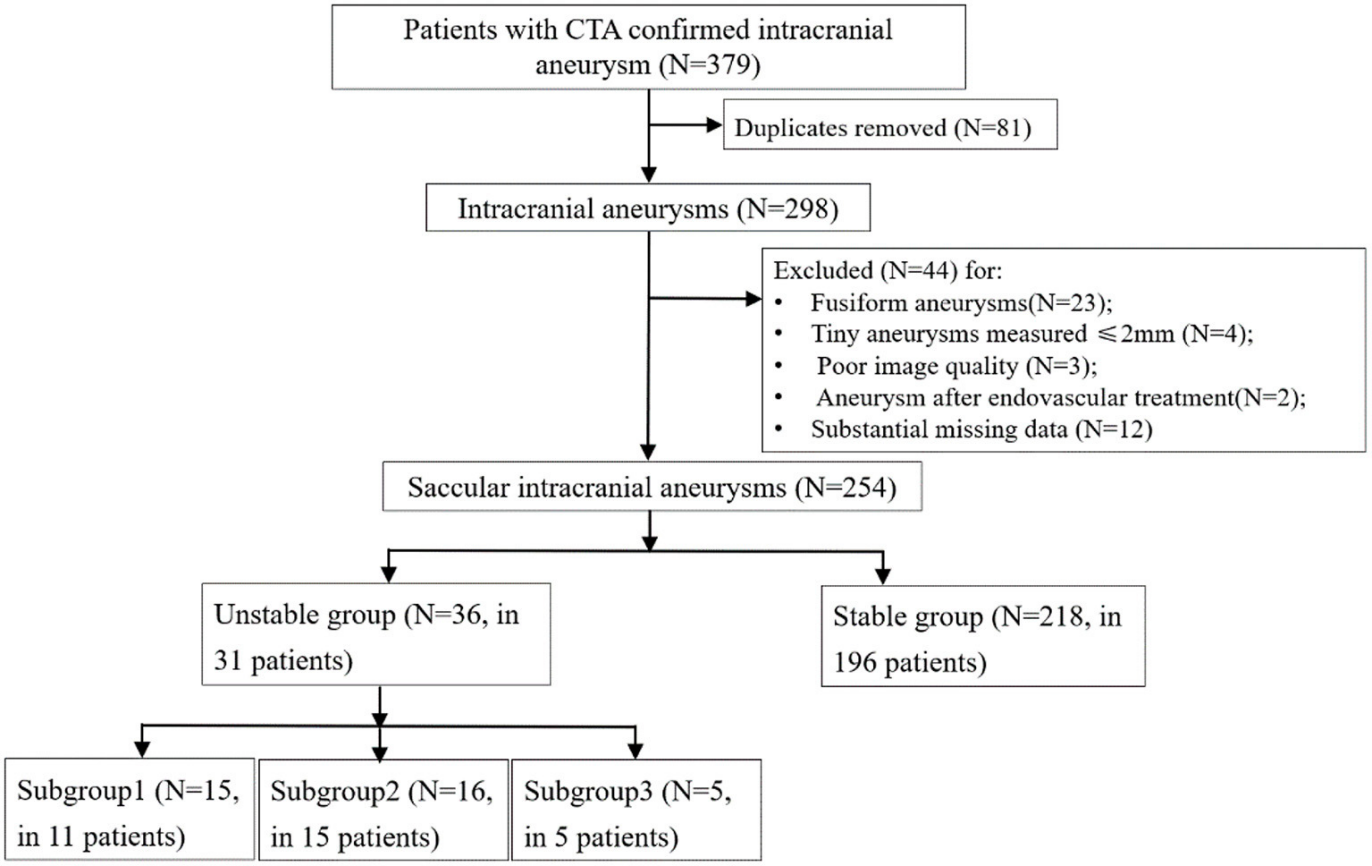
Flowchart of the patients' inclusion and exclusion process. Subgroup1, aneurysms with a ruptured post-imaging; Subgroup2, aneurysms with growth on serial imaging; Subgroup3, aneurysms with compressive symptoms.

A flowchart of the patients' inclusion and exclusion process was shown in Figure 1.

### Clinical Data

Demographic and clinical information were collected from the medical record which included age, sex, history of hypertension, hyperlipidemia, diabetes, smoking and alcohol use. Hypertension is diagnosed if the mean blood pressure at rest is ≥130 mmHg systolic or ≥80 mmHg diastolic. Hyperlipidemia is generally diagnosed at a fasting serum (or plasma) TG (triglyceride) level of ≥150 mg/dL (1.7 mmol/L). FPG (fasting plasma glucose) values ≥126 mg/dL (7.0 mmol/L) can be diagnosed as diabetes, where fasting is defined as no caloric intake for at least 8 h.

### Imaging Techniques

The imaging protocol included standard CT angiography of the head or head and neck acquired on SOMATOM Definition AS + (Siemens Healthineers, Erlangen, Germany) from Sichuan Provincial People's Hospital. The scans employed the following parameters: kV, 100; mA, 250–300; section thickness, 0.75 mm; reconstruction interval, 0.5 mm; scanning direction, from the second cervical vertebra to the vertex. Iohexol non-ionic contrast agent (Shanghai General Electric Pharmaceutical Co., Ltd, Shanghai, China) was injected through elbow vein with double syringe at an injection rate of 4.5 ml/s. An automatic fluoroscopic bolus–triggered system was set to determine the timing of data acquisition. The scanning was started when contrast agent concentration reached threshold levels at the C2-C3 level of the internal carotid artery.

### Extraction of Traditional Morphological Features

Traditional morphological features identified from the included CTA images were as follows:

Dmax (Maximum diameter) was defined as the largest distance between any two points along the surface of the aneurysm.

Shape was considered as regular or irregular (aneurysms with daughter sacs, multiple lobes, or other types of wall protrusions).

Dn (Neck diameter) was computed as twice the average distance from the neck centroid to the neck border.

Height (Aneurysm maximum height) was defined as the largest distance between the center of the neck (geometric center) and the aneurysm surface.

Dv (Parent vessel diameter) was computed as twice the average distance from the parent vessel cross section centroid to the parent vessel plane border.

AR (Aspect ratio) was defined as the ratio between Maximum height and Neck diameter.

SR (Size ratio) was defined as the ratio between Maximum height and Parent vessel diameter (23).

Location of aneurysms was divided into 4 categories: intracranial intradural carotid artery and posterior communicating artery, middle cerebral artery, anterior cerebral artery circulation (anterior cerebral artery and anterior communicating artery), and posterior circulation (vertebral artery, basilar artery, and posterior cerebral artery).

All features of IAs were evaluated and measured by two raters (R.L. and P.Z.) and confirmed by one senior radiologist with 10 years of clinical imaging experience (Y.W.). Two raters measured all samples, respectively. The inter-rater reliabilities of the traditional morphological features were performed by evaluating intra-class correlation.

### Image Segmentation, Preprocessing, and Radiomics Features Extraction

Accounting for the variation in slice thickness, before feature extraction, B-spline algorithm was used to re-slice the images to standard template of voxels with 1 × 1 × 1 mm size. Radiomics software (Prototype, Siemens-healthineers, Erlangen, Germany) was used for identifying Regions of interest (ROIs). The two raters drew manual ROIs slice-by-slice on the original sagittal, reconstructed axial, and coronal CTA images, outlining the aneurysm boundary to cover the entire lesion volume. Each rater drew half the samples in order independently and an additional 20% of the patients for reliability testing. ROIs included as few peripheral structures (adjacent blood vessels or soft tissues) as possible. After the ROI segmentation was completed, Radiomics features were extracted automatically by using the calculation function of the software, and the features were extracted according to the feature guidelines of the Image Biomarker Standardization Initiative (IBSI) (24).

First-order statistics, shape (2D), shape (3D), gray-level co-occurrence matrix (GLCM), gray-level size-zone matrix (GLSZM), gray-level run-length matrix (GLRLM), neighborhood gray-tone difference matrix (NGTDM), and neighboring gray-level dependence matrix (GLDM) were the eight feature groups that were extracted. Advanced filters including Laplacian of Gaussian (LoG; sigma, 2.0 and 3.0 mm), wavelet decompositions with all possible combinations of high- (H) or low- (L) pass filter in each of the three dimensions (HHH, HHL, HLH, LHH, LLL, LLH, LHL, HLL), and local binary pattern (LBP; level, 2; radius, 1.00) were also applied. Definitions and calculations of the Radiomics features used in this study can be found in the PyRadiomics documentation (24, 25).

### Model Construction and Evaluation

Stratified random sampling technique was applied to create two random samples of training and independent testing sets within studied classes. Given the relatively low percentage of unstable aneurysms (36/254 = 14.2%), we used a ratio of 1:1 for samples size allocation for two sets. If the training vs. test ratio was larger, such as 6:4 or even 7:3, the sensitivity and specificity of the model would swing disproportionately by the misjudgment of one single positive case in the test set. In consideration of this issue, increasing the proportion of test sets was suggested from a statistic perspective (26).

The training set was used to train the model based on least absolute shrinkage and selection operator (LASSO) regression model with tuning parameter of lambda. Since there was class imbalance that might affect the model tuning, synthetic minority over-sampling technique (SMOTE) was applied to the training sets before formal model training. The lambda was tuned across multiple values between 0.01 and 2. The optimal value of lambda was decided when the model gained the highest AUC based on 5 repeats 10-folds cross-validation. Subsequently, the importance of the features based on this model with optimal lambda were ranked. Next the conventional logistic multivariate regression model was fitted. To ensure both the inferential and prediction performance of this statistical model, we only enrolled a limited number of features based on the above mentioned importance rank list. The number was decided by obeying the rule suggested by Green (27), that *N* ≥ 50 + 8 m, where m is the number of features and *N* is the number of samples.

The model performance was evaluated in training and testing sets, respectively. These two apparent performances were shown in terms of receiver operator curves. Sensitivity, specificity, and accuracy were also illustrated using a confusion matrix. In addition, to allow for the estimation of the model generalizability, bootstrapping resampling methods were applied to SMOTE training sets to generate 5 samples for model evaluation.

Three basic prediction models were built using selected features to predict aneurysm stability. Model A was constructed using traditional morphological parameters measured directly from images. Model B was based on the selected Radiomics features calculated by the software. Among different categories of Radiomics features, morphological features, especially advanced ones, such as flatness and elongation, have gained considerable attention in the literature as potential novel biomarkers for aneurysm stability (18). In order to specifically assess the performance of these features, Model C was constructed with only Radiomics features related to morphology. It has been argued that the utility of grayscale image characteristics and texture parameters in Radiomics features are limited in assessing intracranial aneurysms, and morphological parameters are more meaningful (18). Therefore, we separately analyzed and verified the morphological parameters in Radiomics by Model C in addition to the Radiomics model, Model B. Integrated models of traditional and Radiomics features were also built (model A+B, model A+C).

Model A, model B, model C and integrated models were evaluated and compared by calculating the AUC on both the training and test sets.

### Statistical Analysis

The Shapiro-wilk test was used to test the normality of continuous variables. Continuous variables were summarized as means ± standard deviation if normally distributed, or median and interquartile ranges (IQRs) if otherwise. Categorical variables were presented as percentages. Fisher exact test or chi-square test was used for comparisons between groups for categorical variables, and Mann-Whitney *U*-test was used for continuous variables. Univariate and multivariate analyses were performed sequentially. Binary logistic regression was performed with a backward stepwise method. Correlation of variables was assessed by Pearson Correlation Coefficient for normally distributed continuous variables, and by Spearman's Rank-Order Correlation otherwise. The statistical significance was defined as *p* < 0.05 (two-tailed). To assess inter-rater variability, we used the intra-class correlation coefficient (ICC) for each feature (two-way random effects, single rater/measurement, absolute agreement, with the value higher than 0.9 regarded as excellent). For statistical analyses, SPSS (Statistics Version 26, Chicago, IL, United States) was used.

## Results

### Patients and Aneurysm Characteristics

A total of 227 patients [149 (65.6%) females] with 254 intracranial aneurysms met the criteria and were included. There were 31 (13.7%) patients with 36 (14.2%) unstable aneurysms in the positive group. Fifteen of the 35 unstable aneurysms had a near-term rupture event, 16 had growth in sequential imaging follow-up (median, 6.5 months; range, 0.5–35 months), and 5 had compressive symptoms, respectively. The demographic, clinical, and imaging characteristics of the unstable and stable groups are shown in Table 1. No significant differences were present in patients' age, sex, history of hypertension, hyperlipidemia, diabetes, smoking and alcohol use between the two groups (all *p* > 0.05). After splitting, there were 120 patients allocated to the training set, with the remaining cases placed in the testing set. Considering the training set sample size, we could enroll 8 features maximum for each model.

**Table 1:** Traditional morphological characteristics of included patients and aneurysms.

**Variable**	**Total**	**Unstable group**	**Stable group**	***p*-value**
No. of patients	227	31 (13.7%)	196 (86.3%)	NA
No. of aneurysms	254	36 (14.2%)	218 (85.8%)	NA
Age in years, median (IQR)	61 (49–68)	62 (53–67)	58 (48–67)	0.26
Female gender	149 (65.6%)	23 (74.0%)	126 (64.3%)	0.38
Hypertension	50/95 (52.6%)	7/9 (77.8%)	43/86 (50.0%)	0.11
Hyperlipidemia	8/95 (8.4%)	1/9 (11.1%)	7/86 (8.1%)	0.76
Diabetes	9/85 (10.6%)	1/9 (11.1%)	8/76 (10.5%)	0.94
Smoking	29/114 (25.4%)	3/9 (33.3%)	26/105 (24.8%)	0.57
Alcohol use	26/94 (27.7%)	3/9 (33.3%)	23/85 (27.1%)	0.27
AR	1.21 ± 0.70	1.79 ± 0.71	1.11 ± 0.65	<0.01
SR	1.61 ± 1.13	2.07 ± 1.36	1.53 ± 1.01	<0.01
Dn, median (IQR, mm)	2.8 (2.3–3.8)	2.1 (1.7–2.7)	3.0 (2.4–3.9)	<0.01
H, median (IQR, mm)	3.0 (2.4–4.0)	3.6 (2.5–4.7)	3.0 (2.2–3.9)	0.22
Dmax, median (IQR, mm)	3.9 (3.1–5.5)	4.3 (2.8–5.2)	5.0 (3.1–5.6)	0.03
Dv, median (IQR, mm)	2.4 (1.9–2.8)	2.2 (1.6–2.6)	2.4 (2.0–2.8)	0.84
**Aneurysm location**			
ICA/PCOM	190	23 (63.9%)	167 (76.5%)	0.31
AC	21	3 (8.3%)	18 (8.3%)	
PC	8	2 (5.6%)	6 (2.8%)	
MCA	35	8 (22.2%)	27 (12.4%)	

*NO, Number; IQR, Inter Quartile Range; SR, Size ratio; Dv, Parent vessel diameter; Dmax, The maximal diameter of the aneurysm; AC, anterior circulation (anterior cerebral artery and anterior communicating artery); MCA, Middle cerebral artery; AR, Aspect ratio; Dn, Aneurysm neck diameter; H, Hight; ICA/PCOM, intracranial intradural carotid artery and posterior communicating artery; PC, posterior circulation (vertebral artery, basilar artery, and posterior communicating artery)*.

### Inter-Rater and Intra-Rater Agreement

Inter-rater reliability for traditional morphology measurements was found to be good (mean ICC = 0.969; range 0.952–0.987).

A total of 1,691 Radiomics features were extracted and included in the intra-class correlation evaluation. Features with ICC of <0.9 were excluded. A total of 460 features were excluded for unsatisfactory inter-rater agreement. Ultimately, 1,231 features were selected for further analysis. The overall inter-rater agreement of the 1,231 features was excellent (mean ICC = 0.966; range 0.900–1.000).

### Construction and Evaluation of Traditional and Radiomic Models

#### Model A-Traditional Morphological Features Model

Traditional morphological features that were significantly different between the positive and negative groups were employed in this model. The formula of model A is provided in Equation (1):


(1) $$\begin{array}{l} Model\;A\;score\\ =\left(1.336\right)\times AR+\left(2.113\right)\times Dmax+\left(1.753\right)\\ \times Height+\left(-0.480\right)\times Dv-1.474 \end{array}$$

Model A showed good performance in the classification of aneurysm stability [AUC: 0.921 (95% CI: 0.862–0.981) and 0.909 (95% CI: 0.853–0.965) on training and testing sets, respectively]. On the training set, the accuracy, sensitivity and specificity were 85.7, 82.4, and 86.2%, respectively. On the testing set, the accuracy, sensitivity and specificity were 85.9, 77.8, and 87.3%, respectively.

**Figure 2:**
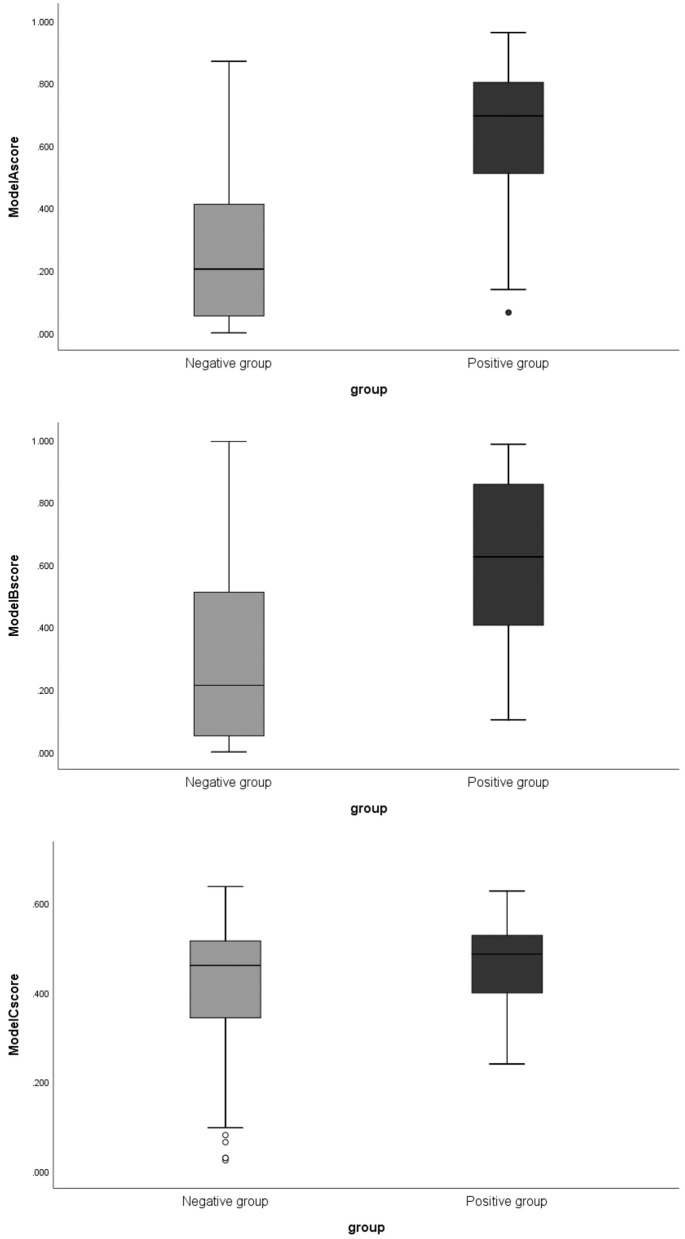
The boxplots of corresponding scores of 3 models comparing negative and positive groups. Model A, model of traditional morphological features; Model B, model of Radiomics derived features; Model C, model of Radiomics derived morphological features.

The boxplots of the scores (The score of each patient on training and testing sets showed the association of high scores with the risk of aneurysm stability.) for the positive and negative groups based on Model A are illustrated in Figure 2. The scores of the negative group were 0.204 (0.05–0.419), and the scores of the positive group were 0.695 (0.484–0.812), (*p* < 0.01). There was little overlap between the two groups of boxplots.

#### Model B-Radiomics Derived Features Model

**Figure 3:**
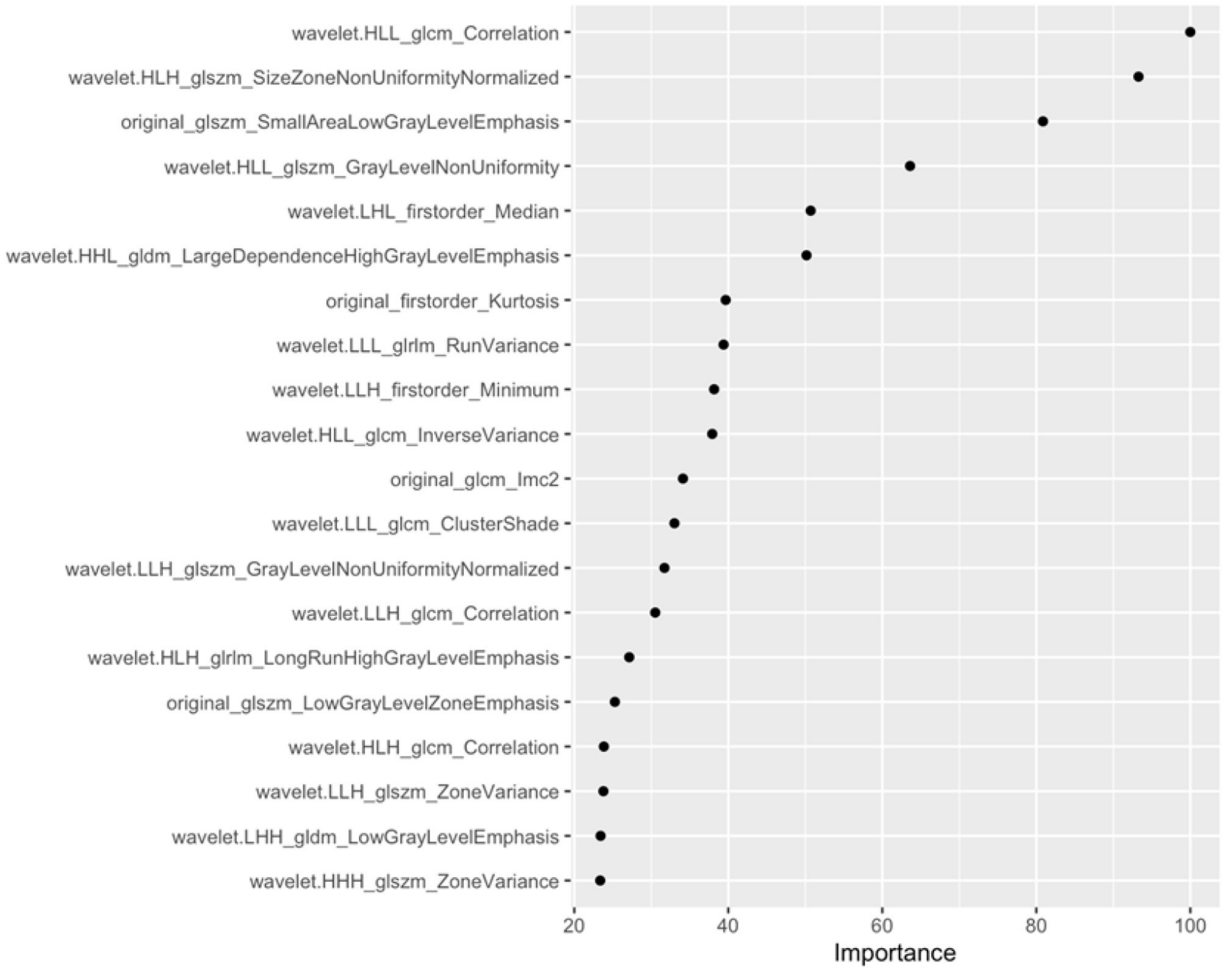
Top 20 features and feature coefficients (feature importance) in the Radiomics model (model B).

Of the 1,231 features included in the analysis, 853 features were significantly different between the positive and negative groups. After enrolling these features into LASSO, the lambda was decided as 0.01 through the tuning process for features importance ranking. And the top 8 features on this list were included in the final conventional logistic regression model. Ultimately, only the first 6 features showing statistical significance (Figure 3) were used to build Model B. And the odds ratio and 95% confidence interval of these features are showed in Table 2. The formula for Model B is provided in Equation (2):


(2) $$\begin{array}{l} Rad\;score\\ =\;(1.576)\times wavelet.HLL\_glcm\_Correlation+(-0.904)\\ \times \;wavelet.HLH\_glszm\_SizeZoneNonUniformityNormalized\\ +(1.952)\times original\_glszm\_SmallAreaLowGrayLevelEmphasis\\ +(0.693)\times wavelet.HLL\_glszm\_GrayLevelNonUniformity\\ +(-0.689)\times wavelet.LHL\_firstorder\_Median+(-1.663)\\ \times \;wavelet.HHL\_gldm\_LargeDependenceHighGrayLevelEmphasis\\ -\;1.262 \end{array}$$

Five of the six features were wavelet characteristics, and the corresponding connotations can be found in the PyRadiomics documentation (24, 25). The performance of Model B in classification of aneurysm stability: AUC: 0.865 (95% CI: 0.778–0.951) on the training set and 0.739 (95% CI: 0.636–0.841) on the testing set. On the training set, the accuracy, sensitivity and specificity were 77.3, 82.4, and 76.5%, respectively. On the testing set, the accuracy, sensitivity and specificity were 71.7, 61.1, and 73.5%, respectively.

**Table 2:** Six Radiomics features that were screened out to build model B.

**Feature**	***p*-value**	**OR**	**95% CI for OR**
wavelet.HLL_glcm_Correlation	<0.0001	4.837	2.566–9.117
wavelet.HLH_glszm_SizeZoneNonUniformityNormalized	0.0007	0.405	0.240–0.685
original_glszm_SmallAreaLowGrayLevelEmphasis	<0.0001	7.041	3.624–13.682
wavelet.HLL_glszm_GrayLevelNonUniformity	0.0224	2.000	1.103–3.626
wavelet.LHL_firstorder_Median	0.0043	0.592	0.313–0.806
wavelet.HHL_gldm_LargeDependenceHighGrayLevelEmphasis	<0.0001	0.190	0.088–0.408

*OR, odds ratio; 95% CI, 95% confidence interval*.

The boxplot of the scores for the positive and negative groups based on Model B are illustrated in Figure 2. The scores were 0.214 (0.054–0.519) in the negative group and 0.695 (0.398–0.8865) in positive group (*p* < 0.01).

#### Model C-Radiomics Derived Morphological Features Model

There were three Radiomics derived morphological features that showed significant difference after analysis, which were original_shape_Maximum2DDiameterSlice, original_shape_LeastAxisLength and Flatness. The formula for model C is provided in Equation (3):


(3) $$\begin{array}{l} Model\;C\;score\\ =\left(1.260\right)\times original\_shape\_Maximum2DDiameterSlice\\ +\left(-1.426\right)\times original\_shape\_LeastAxisLength\\ +\left(0.583\right)\times Flatness-0.347 \end{array}$$

Model C showed a barely satisfactory performance in classification of aneurysm stability [AUC: 0.605 (95% CI: 0.470–0.739) on the training set and 0.552 (95% CI: 0.401–0.703) on the testing set.] On the training set, the accuracy, sensitivity, and specificity were 61.7, 44.4, and 64.5%, respectively. On the testing set, the accuracy, sensitivity and specificity were 61.9, 41.2, and 65.1%, respectively.

The boxplot of the scores for the positive and negative groups based on Model C are illustrated in Figure 2. The scores were 0.461 (0.342–0.516) for the negative group and 0.486 (0.399–0.599) for the positive group (*p* = 0.073).

**Figure 4:**
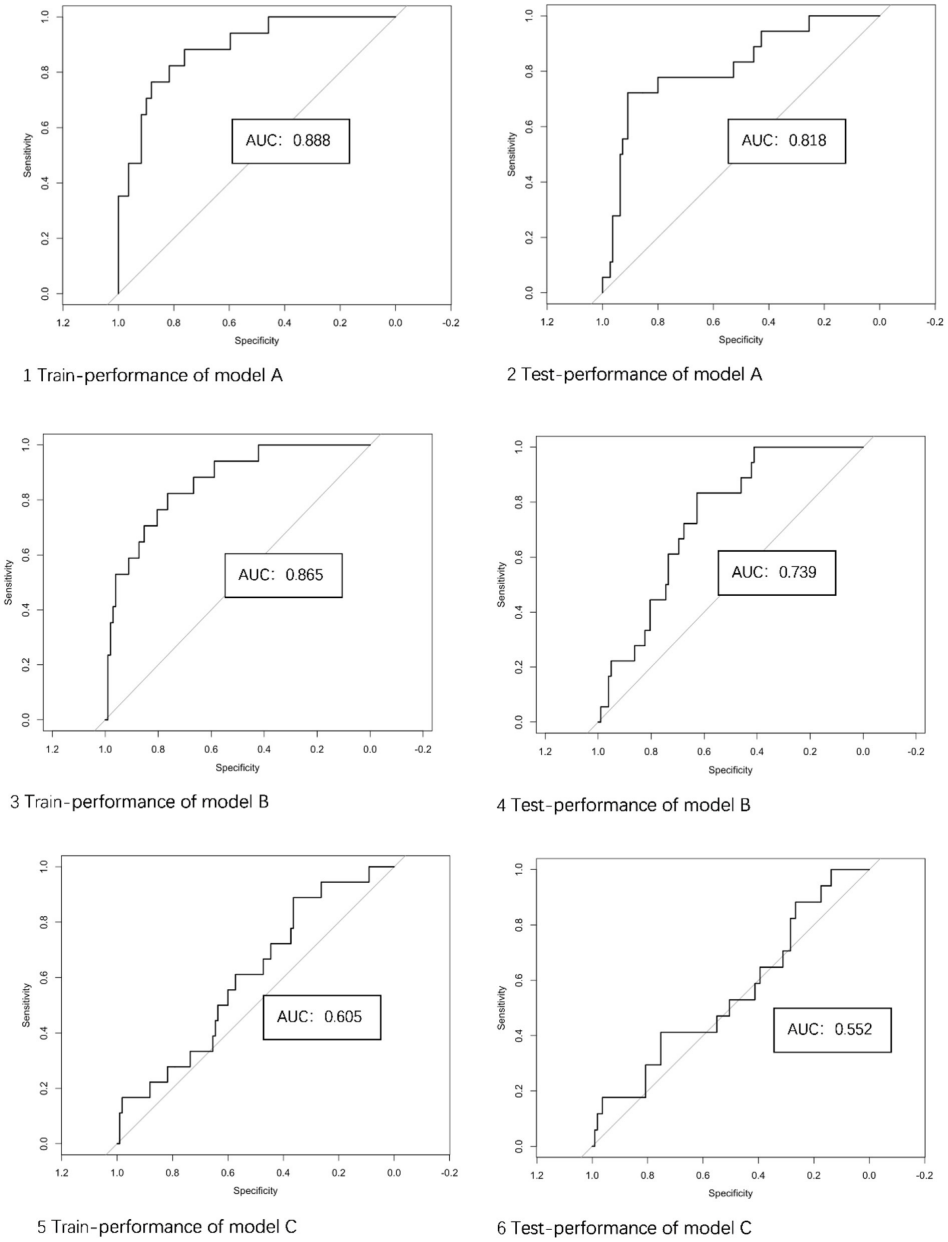
The performance of 3 models in the training and testing sets. AUC, area under the curve; Model A, model of traditional morphological features; Model B, model of Radiomics derived features; Model C, model of Radiomics derived morphological features.

The ROC curves of the 3 models in the training and testing sets are illustrated in Figure 4. The performance of model C was lower than that of model A or model B, and model B was not superior to model A. The performance of the 3 models for training and testing sets is presented in Table 3.

**Table 3:** Diagnostic performance of models in training and testing set.

**Model types**	**Set types**	**AUC** **(95%CI)**	**Accuracy** **(%)**	**Sensitivity** **(%)**	**Specificity** **(%)**
Model A	Training	0.888 (0.808–0.967)	84.1	76.5	85.3
	Testing	0.818 (0.705–0.932)	81.2	72.2	82.7
Model B	Training	0.865 (0.777–0.952)	77.3	82.4	76.5
	Testing	0.739 (0.636–0.841)	71.7	61.1	73.5
Model C	Training	0.605 (0.470–0.740)	61.7	44.4	64.5
	Testing	0.551 (0.401–0.703)	61.9	41.2	65.1

*AUC, area under the curve; CI, confidence interval; Model A, model of traditional morphological features; Model B, model of Radiomics derived features; Model C, model of Radiomics derived morphological features*.

**Figure 5:**
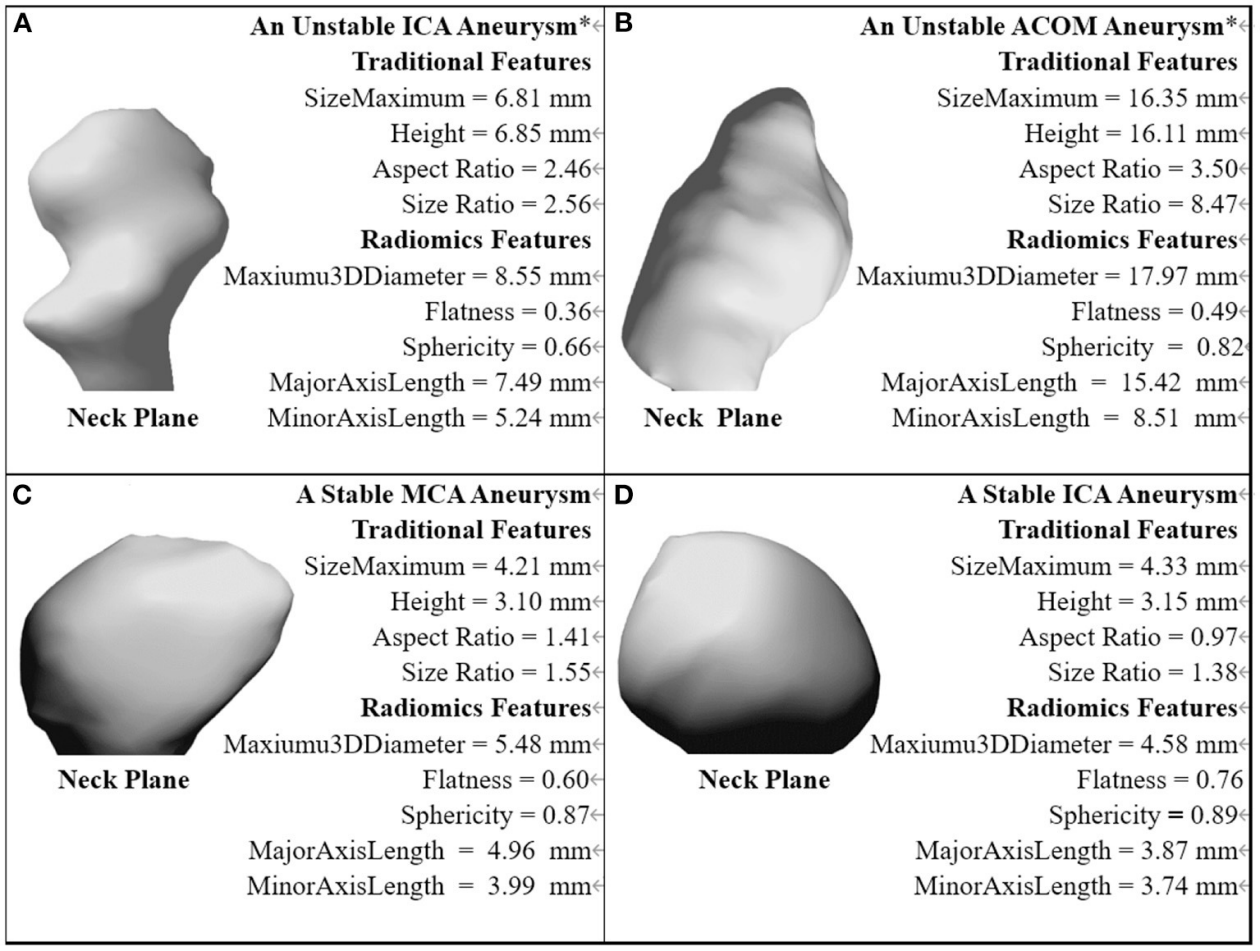
The traditional and Radiomics features of demonstrative cases of four categories. *The aneurysm with a near-term rupture event.

The traditional and Radiomics features of demonstrative cases of four categories are illustrated in Figure 5.

### The Integrated Model

**Figure 6:**
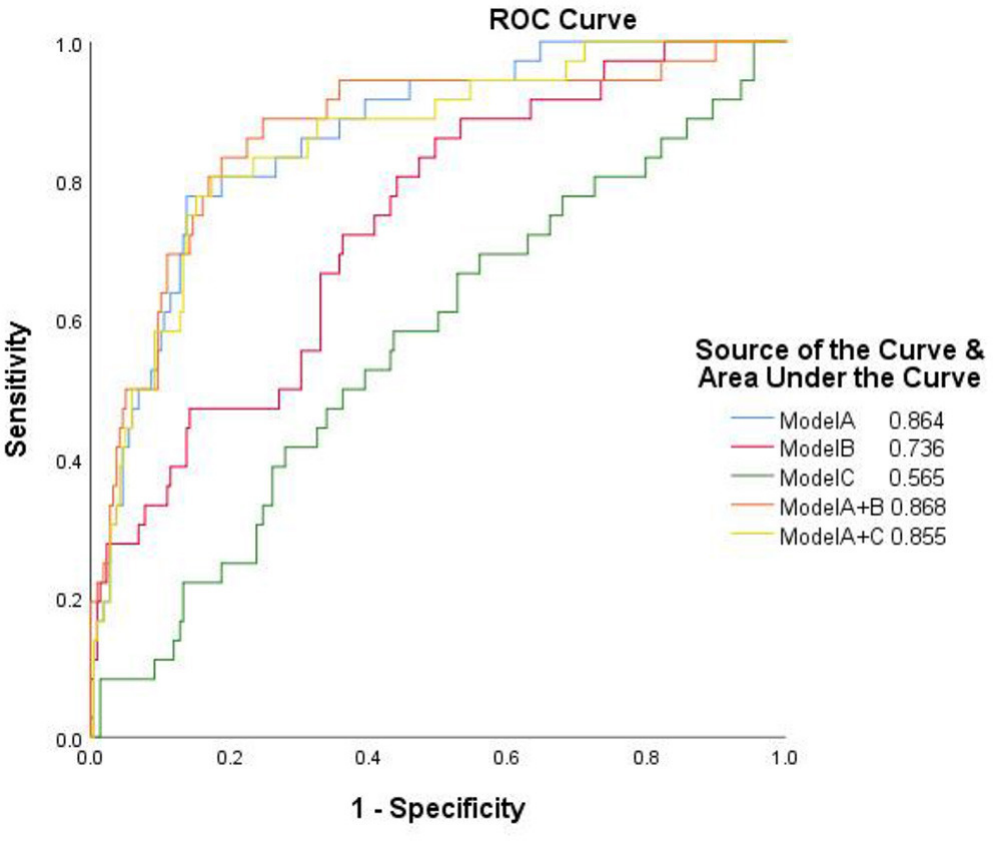
The receiver operator characteristic curves of the separate and integrated models. Model A, model of traditional morphological features; Model B, model of Radiomics derived features; Model C, model of Radiomics derived morphological features.

The performance of the integrated models of traditional and Radiomics features as evaluated by ROC are shown in Figure 6. The AUC value of the integrated model A+B was 0.868, which was slightly higher than any single model, but not statistically significant. The integrated model A+C failed to improve diagnostic performance in AUC compared to model A.

### Correlation Between Flatness and Traditional Morphological Features

**Figure 7:**
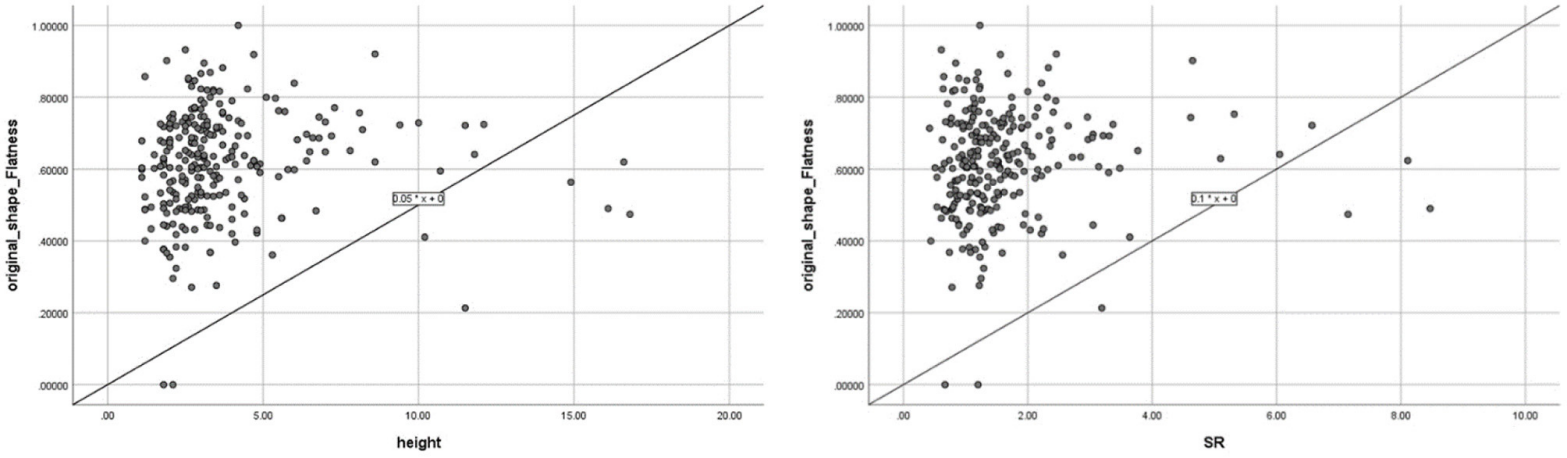
Correlation between Flatness and the two traditional morphological features (height and size ratio, which showed significant correlations with Flatness).

Flatness was the only advanced morphological feature among the selected features. The relationship between flatness and traditional morphological features was evaluated as follows: among traditional morphological features, including Height, Dmax, Dn, Dv, AR, and SR, Flatness correlated best with Height (*p* < 0.01, *R*^2^ = 0.19), followed by SR (*p* < 0.05, *R*^2^ = 0.13), as shown in Figure 7. The correlation between flatness and each traditional morphological feature is shown in Table 4.

**Table 4:** Correlation between the advanced morphological feature flatness and traditional features.

		**Dn**	**Dv**	**AR**	**SR**	**Height**	**Dmax**
original_shape_Flatness	Correlation coefficient	0.08	0.114	0.113	0.128*	0.188**	0.102
	*p-*value	0.202	0.07	0.073	0.042	0.003	0.105

* *p < 0.05*.

** *p < 0.01*.

## Discussion

In this study, multiple models (Radiomics-inclusive, Radiomics derived morphological, traditional morphological) based on CTA images were constructed and compared to identify features associated with aneurysm stability. Significant differences in both Radiomics features and traditional aneurysm morphology between stable and unstable aneurysms were found. Most characteristics of building model B were wavelet features. Wavelet features were the intensity and texture features of the original image obtained by wavelet decomposition calculation. The features were concentrated in different frequency ranges within the aneurysm volume, which could reflect the blood flow in the aneurysm. The difference between the stable group and the unstable group may come from the turbulent state in the unstable aneurysm. Although the model based on Radiomics features could predict aneurysm stability at a relatively high level in both training and testing sets (AUCs of 0.865 and 0.739, respectively), it did not appear superior to the model based on traditional morphological features (AUCs of 0.888 and 0.818, respectively). The AUC of the model integrating Radiomics and traditional morphological features appeared not significantly higher, whereas the performance of Radiomics-derived morphological features alone was not satisfactory.

Our results showed that Flatness was the only significant advanced morphological parameter derived by Radiomics to identify aneurysm instability, which is similar to results from Liu et al. using DSA data (18). Ludwig et al. reported that Radiomics-derived Elongation and Flatness were two significant predictors of aneurysm rupture. But they also found the two features were closely related (*R*^2^ = 0.75) (18), which could be the reason that the two features were not preserved simultaneously in multivariate analysis in this study. We also found that Flatness was correlated to some traditional morphological parameters, most strongly with Height (*R*^2^ = 0.19) and SR (*R*^2^ = 0.13).

Some clinical risk factors are considered to be related to the development, growth, and rupture of intracranial aneurysms, such as age, sex, race, family history, hypertension history, smoking history, drinking history, and previous stroke history of patients (17, 28). The results of each study are different, mainly due to the differences of included study populations (17, 29). No significant differences were present in these clinical risk factors between the two groups in this study, probably due to the study population.

### Comparison Between Advanced Features and Traditional Morphological Features

Our results show that the model performance based on traditional morphological features is still good (AUCs of 0.888 and 0.818, respectively in training and testing sets). By comparison, the Model A score had little overlap between the two groups, as shown in the box plots (Figure 2). Features defined by ratios such as AR were shown as strong factors related to aneurysm rupture [also supported by our previous large-sample study of a similar population (30)], which may not be fully presented in the definition of Radiomics features. A recent study using machine learning to classify intracranial aneurysm rupture status on CTA also screened out mainly traditional morphological features, such as irregular shape and size ratio as the most important discriminators (31). Another study also revealed that Radiomics were not obviously superior to traditional morphological features (18), which is consistent with our study. Although traditional morphological features and Radiomics features can be integrated, only the model integrating traditional features and overall Radiomics features (model A+B) showed a marginal improvement in performance. The use of Radiomics-derived morphological features alone (Model C), however, showed poor performance.

### Context and Novelty

This study has several novel features compared to previous studies. The study by Liu et al. was based on DSA (12), which is invasive and not used for screening and re-examination. CTA was used in this study as it is the most frequently employed imaging modality for aneurysm evaluation. Previous studies only assessed the region of interest from a single image slice (32), while we delineated the region of interest from all image slices with a slice thickness of 0.75 mm, and extracted three-dimensional aneurysm volumes as the basis of radiomic and morphological characteristic analysis.

Two previous Radiomics studies based on CTA images aimed to distinguish ruptured and unruptured aneurysms, considering already ruptured aneurysms as unstable ones (18, 19). Aneurysm morphology, however, may change after rupture (14), and the identified biomarkers and corresponding conclusions may not be directly applied to predict the risk in the group of unruptured aneurysms. In this study, more comprehensive and representative criteria of aneurysm instability were used, and all the included aneurysms were considered to have not yet ruptured at the time of CTA imaging. The conclusions can therefore be more directly interpreted and used clinically to predict rupture risk.

As reported, the proportion of aneurysm growth in follow-up studies was relatively low (mostly reported around 5%, one study reported 12% in 2 years) (33–35). Aneurysms at near-rupture status are difficult to obtain clinically. In this study, categorized 14.2% (*N* = 36) aneurysms into the unstable group, which reduced the limitations of data imbalance and facilitated model construction. Radiomics calculation is a fast and automatic process using one-station software. Once the structures of interest have been semi-automatically delineated, the selected Radiomics features and the calculation of Rad-score can be readily employed in clinical practice with further investigation and validation.

## Limitations

There were several limitations in our study. First, it was a retrospective analysis. Planned recruitment of patients harboring intracranial aneurysms with sentinel headaches was not conducted. Second, not all cases had follow-up data available, and parts of the clinical demographic data were incomplete, especially for outpatients. But as much data as possible was collected thorough a search of the institutional database. Third, the research was from a single medical center with a small sample size of unstable aneurysms, and external validation of the results could not be obtained. Prospective and multi-center studies with larger numbers of cases could be used for further validation.

## Conclusion

A radiomics and traditional morphology integrated model seems to be an effective tool for identifying intracranial aneurysm instability, whereas the use of Radiomics-derived morphological features alone is not recommended. Radiomics-based models were not superior to the traditional morphological features model.

## Data Availability Statement

The original contributions presented in the study are included in the article/supplementary material, further inquiries can be directed to the corresponding author/s.

## Ethics Statement

This retrospective study was approved by the ethics committee of Sichuan Provincial People's Hospital, and the requirement for written informed consent was waived.

## Author Contributions

YW contributed to conception and design of the study. RL and PZ organized the database. XC performed the statistical analysis. RL wrote the first draft of the manuscript. PZ, XC, and RL wrote sections of the manuscript. All authors contributed to manuscript revision, read, and approved the submitted version.

## Funding

This work was supported by the Scientific Research Fund of Sichuan Provincial People's Hospital, 2020LY05.

## Conflict of Interest

XC was employed by Siemens Healthineers. The remaining authors declare that the research was conducted in the absence of any commercial or financial relationships that could be construed as a potential conflict of interest.

## Publisher's Note

All claims expressed in this article are solely those of the authors and do not necessarily represent those of their affiliated organizations, or those of the publisher, the editors and the reviewers. Any product that may be evaluated in this article, or claim that may be made by its manufacturer, is not guaranteed or endorsed by the publisher.
